# Clinical features and prognosis of patients with and without diabetes mellitus undergoing endovascular aortic aneurysm repair

**DOI:** 10.1186/s12902-022-01008-4

**Published:** 2022-04-07

**Authors:** Mitsuyoshi Takahara, Osamu Iida, Junichi Tazaki, Ryusuke Nishikawa, Kiyonori Nanto, Yoshiro Chiba, Kazuhisa Sakamoto, Makoto Kinoshita, Naoki Takahashi, Satoshi Kamihira, Terutoshi Yamaoka, Hirooki Higami, Takeichiro Nakane, Takahiro Ohmine, Atsushi Guntani

**Affiliations:** 1 Department of Diabetes Care Medicine, Osaka University Graduate School of Medicine, 2-2 Yamadaoka, Suita City, Osaka 565-0871 Japan; 2 Cardiovascular Center, Kansai Rosai Hospital, 3-1-69 Inabaso, Amagasaki City, Hyogo 660-8511 Japan; 3 Department of Cardiovascular Medicine and Department of Cardiovascular Surgery, Graduate School of Medicine, Kyoto University, Yoshida-Konoe-cho, Sakyo-ku, Kyoto City, Kyoto 606-8501 Japan; 4 Department of Cardiovascular Medicine, Shizuoka General Hospital, 4-27-1 Kita Ando Aoi-ku, Shizuoka City, Shizuoka 420-8527 Japan; 5 Department of Cardiology, Mito Saiseikai General Hospital, 3-3-10 Futabadai, Mito City, , Ibaraki 311-4198 Japan; 6 Department of Cardiovascular Medicine, Kobe City Medical Center General Hospital, 2-1-1 Minatojimaminamimachi, Chuo-ku, Kobe-city, Hyogo 650-0047 Japan; 7 Cardiovascular Center, Osaka Red Cross Hospital, 5-30 Fudegasakicho, Tennoji-ku, Osaka City, Osaka 543-8555 Japan; 8 Department of Cardiovascular Surgery, Shimane Prefectural Central Hospital, 4-1-1 Himebara, Izumo City, Shimane 693-8555 Japan; 9 Department of Vascular Surgery, Matsuyama Red Cross Hospital, 1 Bunkyocho, Matsuyama City, Ehime 790-0826 Japan; 10 Department of Cardiovascular Medicine, Japanese Red Cross Otsu Hospital, 1-1-35 Nagara, Otsu City, Shiga 520-0046 Japan; 11 Department of Cardiovascular Surgery, Mitsubishi Kyoto Hospital, 1 Katsuragosho-cho, Nishikyo-ku, Kyoto City, Kyoto 615-8087 Japan; 12 Department of Vascular Surgery, Hiroshima Red Cross Hospital & Atomic-Bomb Survivors Hospital, 1-9-6 Sendamachi, Naka-ku, Hiroshima City, Hiroshima 730-8619 Japan; 13 Department of Vascular Surgery, Saiseikai Yahata General Hospital, 5-9-27 Harunomachi, Yahatahigashi-ku, Kitakyushu City, Fukuoka 805-0050 Japan

**Keywords:** Aortic aneurysm, Endovascular repair, Diabetes mellitus, Clinical profiles, Prognosis

## Abstract

**Background:**

This study aimed to compare the clinical features and prognoses of patients with and without diabetes mellitus (DM) who underwent endovascular repair for aortic aneurysm (AA).

**Methods:**

We analyzed the clinical database of a prospective multicenter study, registering 929 patients who underwent their first endovascular AA repair in Japan between January 2016 and June 2018. The baseline characteristics and prognoses (including all-cause mortality and cardiovascular events) after repair were compared between the DM and non-DM groups. Prognoses were also compared between the groups after propensity score matching.

**Results:**

In total, 226 patients (24.3%) had DM. Compared with non-DM patients, DM patients had higher pack-years of smoking (*P* = 0.011), higher body mass index (*P* = 0.009), lower high-density lipoprotein cholesterol levels (*P* = 0.038), higher triglyceride levels (*P* = 0.025), and lower left ventricular ejection fraction (*P* = 0.005). Meanwhile, the low-density lipoprotein cholesterol and blood pressure levels showed no significant intergroup difference (all *P* > 0.05). DM patients had a higher prevalence of myocardial infarction (*P* = 0.016), history of coronary revascularization (*P* = 0.015), and lower extremity artery disease (*P* = 0.019). Lesion characteristics and procedures were similar between the groups (all *P* > 0.05). DM patients had a higher risk of all-cause mortality and cardiovascular events than non-DM patients (both *P* < 0.001). Subsequent propensity score matching also demonstrated that DM patients had a significantly lower rate of overall survival (*P* = 0.001) and freedom from cardiovascular events (*P* = 0.010). The Kaplan–Meier estimates at 1 year for the overall survival were 85.6% (95% confidence interval [CI], 80.9% to 90.5%) and 94.3% (95% CI, 91.7% to 97.0%) for patients with and without DM, respectively. The corresponding estimates for freedom from cardiovascular events were 79.8% (95% CI, 74.5% to 85.5%) and 87.7% (95% CI, 84.2% to 91.3%), respectively.

**Conclusions:**

Among patients undergoing endovascular AA repair, those with DM had more cardiovascular risk factors. DM patients had a higher incidence rate of all-cause mortality and cardiovascular events. Matching analysis indicated that DM per se would be a risk factor for poor prognoses after AA repair.

**Supplementary Information:**

The online version contains supplementary material available at 10.1186/s12902-022-01008-4.

## Background

Aortic aneurysms (AAs) are usually asymptomatic; however, ruptures, which may occur suddenly, are often life-threatening [1]. Repair of AA is performed primarily for the prevention of its incident rupture. Repair is conventionally performed by open surgery, but endovascular repair has recently become more common in clinical practice, with accumulating evidence of its efficacy and safety being comparable to open surgery [2–5].

The classic risk factors for atherosclerosis, including smoking, uncontrolled blood pressure (BP), and abnormal lipid profiles, increase AA risk in a dose-dependent manner [6–9]. In contrast, diabetes mellitus (DM) has a protective effect on the development and expansion of AA, and DM patients are less likely to suffer from AA than non-DM patients [10–12]. Nonetheless, in clinical practice, some patients with DM develop AA that requires repair. It is of clinical interest whether their clinical features might be different from those of non-DM patients with AA. However, to date, few detailed data are available, especially regarding smoking amount, BP control, and lipid profiles [13–15].

Another clinical interest is the prognosis of patients with DM after AA repair. While several previous studies have investigated the prognostic impact of DM after AA repair [14–19], none included detailed data on the patients’ baseline characteristics, such as smoking amount, BP control, and lipid profiles. Therefore, their results might be confounded by these unmeasured clinical backgrounds. This study aimed to compare the clinical features and prognoses between patients with and without DM who underwent endovascular repair for AA.

## Methods

The current study analyzed a baseline and 1-year database of the Efficacy and safety Of endovascuLar repair for abdominal and thoracIc aortic Aneurysms (EOLIA) registry. The EOLIA registry is an ongoing prospective multicenter observational study that registered adult patients (≥ 20 years) undergoing their first endovascular repair for AA in Japan. The study subjects were registered between January 2016 and June 2018, and 5-year follow-ups have been scheduled. A total of 929 patients with AA were included in the study. The study was conducted in accordance with principle of the Declaration of Helsinki and was approved by the ethics committees of the participating centers. Informed consent was obtained from the participants or, if not possible, from their families.

### Definitions

DM was determined when fasting plasma glucose levels were ≥ 126 mg/dL, casual plasma glucose levels were ≥ 200 mg/dL, hemoglobin A1c levels were ≥ 6.5%, or patients were treated with anti-diabetic medications [20]. Hypertension was defined as systolic BP ≥ 140 mmHg, diastolic BP ≥ 90 mmHg, and/or treatment for hypertension [21]. Dyslipidemia was defined as low-density lipoprotein (LDL) cholesterol levels ≥ 140 mg/dL, high-density lipoprotein (HDL) cholesterol levels < 40 mg/dL, non-HDL cholesterol levels ≥ 170 mg/dL, triglyceride levels ≥ 150 mg/dL, and/or treatment for dyslipidemia [22]. Body mass index (BMI) was calculated as the weight in kilograms divided by the height in meters squared. Pack-years of smoking were calculated by multiplying the number of packs of cigarettes smoked per day (equal to the number of cigarettes smoked per day divided by 20 cigarettes per pack) by the number of years the person has smoked. Left ventricular ejection fraction (LVEF) was measured using left ventriculography or echocardiography. Lower extremity artery disease (LEAD) was defined as an ankle-brachial index of 0.9 or lower, history of revascularization, and/or major amputation (defined as surgical excision of the limb above the ankle).

The time-to-events assessed in the current study were all-cause mortality and cardiovascular events. Cardiovascular events were defined as a composite of cardiovascular death, aortic events, cerebral events, cardiac events, and lower extremity events. Aortic events included aortic rupture, endoleak, graft occlusion, graft infection, graft migration, sac enlargement (by 5 mm or larger in diameter), and reintervention. Cerebral events included cerebral hemorrhage and cerebral infarction. Cardiac events included myocardial infarction and hospitalization due to heart failure. Lower extremity events included limb-threatening ischemia (defined as ischemic rest pain, ulcer, or gangrene that required revascularization) and major amputation.

### Statistical analysis

Data are presented as mean ± standard deviation (SD) for continuous variables and number (percentage) for categorical variables, unless otherwise mentioned. Statistical significance was set at *P* < 0.05, and 95% confidence intervals (CIs) were reported when appropriate. The differences in baseline characteristics between the DM and non-DM groups were tested using the unpaired *t* test for continuous variables and the chi-square test for categorical variables. Missing data on baseline characteristics were addressed through multiple imputation by the chained equations method. In the procedure, we generated ten imputed datasets and combined the analytic results according to Rubin’s rule. Cumulative incidence rates of time-to-event were estimated using the Kaplan–Meier method, and their inter-population differences were tested using the log-rank test. Hazard ratios of DM for respective time-to-events were calculated using the Cox proportional hazards regression model.

The comparison of the time-to-event incidence between DM and non-DM patients was expected to be interfered with by the inter-population difference in baseline characteristics. Even if DM patients had an apparently higher risk of any events, the increased risk might be due to clinical features characterizing their propensities, rather than the presence of DM per se. Therefore, we subsequently compared the time-to-event incidence after matching the populations based on the clinical features that characterized DM patients. Matching was performed using the propensity score derived from a logistic regression model, with the presence of DM as the dependent variable and baseline characteristics as explanatory variables. The propensity score was separately calculated for each imputed dataset and then averaged across the ten imputed datasets [23]. Matching was based on the logit of the propensity score, within a caliper of 0.2 SD of the value. To maximize the statistical power to detect intergroup prognostic differences, we extracted as many matched non-DM patients to one DM patient as possible. The balance of baseline characteristics after matching was assessed using the absolute standardized differences. The comparison after matching was performed using stratification by pairs, and weighted descriptive statistics are reported.

We additionally investigated the association of plasma glucose and hemoglobin A1c (HbA1c) levels with prognoses in patients with DM, using the Cox proportional hazards regression model. Furthermore, we examined the association of BMI with prognosis in matched non-DM and DM patients. In the analysis, their difference was assessed with the interaction term of BMI and DM in the Cox proportional hazards regression model stratified by matched pairs.

All statistical analyses were performed using R version 4.1.1 (R Development Core Team, Vienna, Austria).

## Results

The baseline characteristics of the study population are summarized in Table 1 and Additional file 1: Tables S1. The mean age was 76 ± 10 years, and 80.5% of the patients were men. A total of 226 patients (24.3%) had DM. In patients with DM, mean plasma glucose levels were 8.4 ± 3.4 mmol/L (data missing, *n* = 3), and mean HbA1c levels were 6.7 ± 1.0% (49 ± 11 mmol/mol) (data missing, *n* = 19).

**Table 1:** Baseline characteristics of overall population

	Overall population	Missing data	Non-DM patients (*n* = 703)	DM patients (*n* = 226)	*P* value
Age (years)	76 ± 10		76 [75 to 77]	75 [74 to 76]	0.065
Male sex	748 (80.5%)		79.2% [76.2% to 82.2%]	84.5% [79.8% to 89.2%]	0.10
Smoking		4 (0.4%)			0.18
Never	245 (26.5%)		28.0% [24.6% to 31.3%]	21.8% [16.4% to 27.2%]	
Past	516 (55.8%)		54.4% [50.7% to 58.1%]	60.1% [53.7% to 66.5%]	
Current	164 (17.7%)		17.6% [14.8% to 20.4%]	18.1% [13.1% to 23.2%]	
Pack-years of smoking		43 (4.6%)			0.011
None	245 (27.7%)		28.5% [25.1% to 31.9%]	22.0% [16.6% to 27.5%]	
> 0 and ≤ 20 pack-years	110 (12.4%)		13.1% [10.5% to 15.6%]	11.4% [6.8% to 16.0%]	
> 20 and ≤ 40 pack-years	207 (23.4%)		24.2% [20.9% to 27.4%]	22.5% [16.9% to 28.1%]	
> 40 pack-years	324 (36.6%)		34.3% [30.7% to 37.9%]	44.1% [37.5% to 50.8%]	
BMI					0.009
< 20 kg/m^2^	178 (19.2%)		21.6% [18.6% to 24.7%]	11.5% [7.3% to 15.7%]	
20 to 25 kg/m^2^	495 (53.3%)		51.6% [47.9% to 55.3%]	58.4% [52.0% to 64.8%]	
≥ 25 kg/m^2^	256 (27.6%)		26.7% [23.5% to 30.0%]	30.1% [24.1% to 36.1%]	
Diabetes mellitus	226 (24.3%)				
Hypertension	819 (88.2%)		87.2% [84.7% to 89.7%]	91.2% [87.4% to 94.9%]	0.14
Dyslipidemia	730 (78.6%)		76.0% [72.8% to 79.1%]	86.7% [82.3% to 91.1%]	0.001
Renal failure on dialysis	37 (4.0%)		3.8% [2.4% to 5.3%]	4.4% [1.7% to 7.1%]	0.85
Chronic obstructive pulmonary disease	319 (34.4%)	3 (0.3%)	34.3% [30.8% to 37.8%]	34.5% [28.3% to 40.7%]	1.00
Malignant neoplasm		1 (0.1%)			0.64
None	700 (75.4%)		76.1% [72.9% to 79.3%]	73.3% [67.5% to 79.1%]	
Cured	126 (13.6%)		12.8% [10.3% to 15.3%]	16.0% [11.2% to 20.8%]	
In treatment	102 (11.0%)		11.1% [8.8% to 13.4%]	10.7% [6.7% to 14.8%]	
Family history of aortic aneurysm	40 (4.4%)	14 (1.5%)	4.8% [3.2% to 6.4%]	3.5% [0.9% to 6.1%]	0.58
Myocardial infarction	119 (12.8%)	1 (0.1%)	11.3% [8.9% to 13.6%]	17.7% [12.7% to 22.7%]	0.016
History of coronary revascularization	195 (21.0%)	1 (0.1%)	19.1% [16.2% to 22.0%]	27.0% [21.2% to 32.8%]	0.015
Cerebral hemorrhage	33 (3.6%)	1 (0.1%)	3.4% [2.1% to 4.8%]	4.0% [1.4% to 6.5%]	0.85
Cerebral infarction	107 (11.5%)	1 (0.1%)	10.7% [8.4% to 13.0%]	14.2% [9.6% to 18.8%]	0.19
LEAD	82 (9.2%)	40 (4.3%)	8.5% [6.4% to 10.6%]	14.4% [9.7% to 19.1%]	0.019
Statin use	466 (50.7%)	10 (1.1%)	48.1% [44.4% to 51.8%]	58.4% [51.9% to 64.8%]	0.009
Beta blocker use	355 (38.6%)	9 (1.0%)	37.5% [33.9% to 41.1%]	41.9% [35.5% to 48.4%]	0.26
Renin-angiotensin system inhibitor use	405 (44.0%)	9 (1.0%)	44.4% [40.7% to 48.1%]	42.1% [35.6% to 48.6%]	0.60
Antiplatelet use	404 (44.0%)	10 (1.1%)	42.3% [38.6% to 46.0%]	49.5% [42.9% to 56.1%]	0.073
Anticoagulant use	147 (16.0%)	9 (1.0%)	15.2% [12.5% to 17.8%]	18.3% [13.2% to 23.3%]	0.32
Systolic blood pressure		6 (0.6%)			0.53
< 120 mmHg	245 (26.5%)		26.5% [23.3% to 29.8%]	26.6% [20.8% to 32.4%]	
120 to 140 mmHg	423 (45.8%)		44.8% [41.1% to 48.4%]	48.9% [42.4% to 55.5%]	
140 to 160 mmHg	200 (21.7%)		22.8% [19.7% to 25.9%]	18.2% [13.2% to 23.3%]	
≥ 160 mmHg	55 (6.0%)		5.9% [4.1% to 7.7%]	6.2% [3.1% to 9.3%]	
Diastolic blood pressure		6 (0.6%)			0.74
< 80 mmHg	590 (63.9%)		64.0% [60.5% to 67.6%]	63.6% [57.3% to 69.9%]	
80 to 90 mmHg	212 (23.0%)		23.5% [20.3% to 26.6%]	21.3% [15.9% to 26.6%]	
90 to 100 mmHg	86 (9.3%)		8.5% [6.4% to 10.6%]	12.0% [7.7% to 16.2%]	
≥ 100 mmHg	35 (3.8%)		4.0% [2.5% to 5.4%]	3.1% [0.8% to 5.4%]	
Non-HDL cholesterol		190 (20.5%)			0.47
< 100 mg/dl (< 2.59 mmol/l)	158 (21.4%)		21.4% [18.2% to 24.5%]	26.2% [20.3% to 32.1%]	
100 to 130 mg/dl (2.59 to 3.36 mmol/l)	262 (35.5%)		35.7% [32.0% to 39.4%]	31.9% [25.3% to 38.6%]	
130 to 170 mg/dl (3.36 to 4.40 mmol/l)	239 (32.3%)		32.5% [28.9% to 36.1%]	30.9% [24.4% to 37.4%]	
≥ 170 mg/dl (≥ 4.40 mg/dl)	80 (10.8%)		10.4% [8.1% to 12.8%]	10.9% [6.7% to 15.2%]	
LDL cholesterol		67 (7.2%)			0.10
< 70 mg/dl (< 1.81 mmol/l)	105 (12.2%)		11.9% [9.5% to 14.3%]	15.5% [10.4% to 20.6%]	
70 to 100 mg/dl (1.81 to 2.59 mmol/l)	308 (35.7%)		35.3% [31.7% to 38.9%]	37.5% [30.9% to 44.2%]	
100 to 140 mg/dl (2.59 to 3.62 mmol/l)	343 (39.8%)		40.1% [36.5% to 43.8%]	36.0% [29.7% to 42.3%]	
≥ 140 mg/dl (≥ 3.62 mmol/l)	106 (12.3%)		12.7% [10.1% to 15.2%]	11.0% [6.8% to 15.1%]	
HDL cholesterol		94 (10.1%)			0.038
< 40 mg/dl (< 1.03 mmol/l)	206 (24.7%)		23.4% [20.1% to 26.6%]	28.9% [22.7% to 35.1%]	
40 to 50 mg/dl (1.03 to 1.29 mmol/l)	268 (32.1%)		31.2% [27.6% to 34.8%]	34.5% [28.1% to 40.8%]	
50 to 60 mg/dl (1.29 to 1.55 mmol/l)	187 (22.4%)		23.7% [20.4% to 27.0%]	18.4% [13.1% to 23.7%]	
≥ 60 mg/dl (≥ 1.55 mmol/l)	174 (20.8%)		21.7% [18.5% to 24.9%]	18.2% [13.0% to 23.5%]	
Triglycerides		34 (3.7%)			0.006
< 100 mg/dl (< 1.13 mmol/l)	350 (39.1%)		42.0% [38.3% to 45.7%]	31.5% [25.3% to 37.7%]	
100 to 150 mg/dl (1.13 to 1.69 mmol/l)	283 (31.6%)		31.0% [27.5% to 34.5%]	31.9% [25.7% to 38.2%]	
150 to 200 mg/dl (1.69 to 2.26 mmol/l)	137 (15.3%)		13.5% [10.9% to 16.1%]	20.8% [15.3% to 26.2%]	
≥ 200 mg/dl (≥ 2.26 mmol/l)	125 (14.0%)		13.4% [10.9% to 16.0%]	15.8% [11.0% to 20.6%]	
Estimated glomerular filtration rate		3 (0.3%)			0.11
< 15 ml/min/1.73 m^2^	47 (5.1%)		5.0% [3.4% to 6.7%]	5.3% [2.4% to 8.2%]	
15 to 30 ml/min/1.73 m^2^	54 (5.8%)		5.6% [3.9% to 7.3%]	6.6% [3.4% to 9.9%]	
30 to 60 ml/min/1.73 m^2^	416 (44.9%)		43.3% [39.6% to 47.0%]	50.0% [43.5% to 56.5%]	
≥ 60 ml/min/1.73 m^2^	409 (44.2%)		46.1% [42.4% to 49.8%]	38.1% [31.7% to 44.4%]	
LVEF		49 (5.3%)			0.005
< 50%	62 (7.0%)		6.4% [4.6% to 8.3%]	9.1% [5.3% to 12.9%]	
50% to 60%	142 (16.1%)		15.2% [12.5% to 18.0%]	18.4% [13.2% to 23.5%]	
60% to 70%	486 (55.2%)		54.1% [50.3% to 57.9%]	58.0% [51.4% to 64.6%]	
≥ 70%	190 (21.6%)		24.2% [21.0% to 27.5%]	14.6% [9.8% to 19.4%]	

Data in non-DM and DM patients are estimated means or proportions [95% confidence intervals] obtained from the multiple imputation

*BMI* Body mass index, *HDL* High-density lipoprotein, *LDL* Low-density lipoprotein, *LEAD* Lower extremity artery disease, *LVEF* Left ventricular ejection fraction

As demonstrated in Table 1, DM patients had higher BMI than non-DM patients (*P* = 0.009). Furthermore, DM patients had higher pack-years of smoking (*P* = 0.011), although smoking status was not significantly different (*P* = 0.18). DM patients also had a higher prevalence of dyslipidemia (*P* = 0.001), myocardial infarction (*P* = 0.016), coronary revascularization history (*P* = 0.015), and LEAD (*P* = 0.019). DM patients received statin therapy more often (*P* = 0.009), achieving similar non-HDL and LDL cholesterol levels (*P* = 0.47 and 0.10), while they had lower HDL cholesterol levels (*P* = 0.038) and higher triglyceride levels (*P* = 0.025). Another difference was LVEF, which was lower in patients with DM (*P* = 0.005). The prevalence of hypertension and BP control were not significantly different (all *P* > 0.05). Lesion characteristics and procedures were similar between patients with and without DM (all *P* > 0.05) (Additional file 1: Tables S1).

**Fig. 1:**
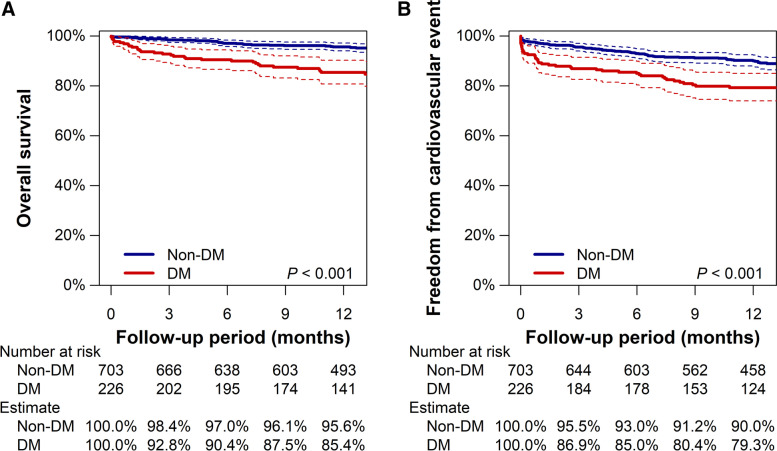
Kaplan–Meier estimates of overall survival (**A**) and freedom from cardiovascular event (**B**) in overall population. Dotted lines represent 95% confidence intervals (CIs)

Figure 1 illustrates the crude prognoses of patients with and without DM. DM patients had a lower rate of overall survival and freedom from cardiovascular events than non-DM patients (both *P* < 0.001). The Kaplan–Meier estimates at 1 year for overall survival were 85.4% (95% CI, 80.8% to 90.3%) and 95.6% (95% CI, 94.0% to 97.2%) for patients with and without DM, respectively. Meanwhile, freedom from cardiovascular events were estimated to be 79.3% (95% CI, 74.0% to 85.0%) and 90.0% (95% CI, 87.7% to 92.3%), respectively.

**Fig. 2:**
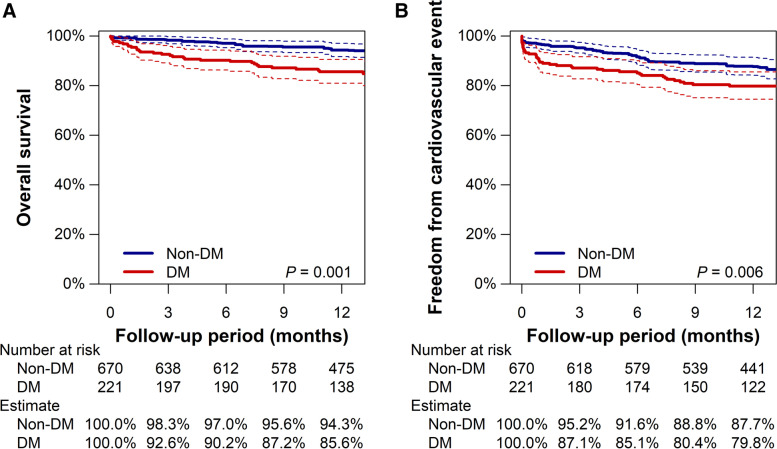
Kaplan–Meier estimates of overall survival (**A**) and freedom from cardiovascular event (**B**) in matched population. Dotted lines represent 95% confidence intervals (CIs)

The subsequent propensity score matching extracted 221 pairs (221 DM patients and 670 non-DM patients). There was no remarkable intergroup difference in baseline characteristics (Table 2 and Additional file 1: Tables S2). In the matched population, DM patients still had a significantly lower rate of overall survival (*P* = 0.001) and freedom from cardiovascular events (*P* = 0.010), as shown in Fig. 2. The Kaplan–Meier estimates at 1 year for the overall survival were 85.6% (95% CI, 80.9% to 90.5%) and 94.3% (95% CI, 91.7% to 97.0%) for patients with and without DM, respectively. Meanwhile, the estimated rates for freedom from cardiovascular events were 79.8% (95% CI, 74.5% to 85.5%) and 87.7% (95% CI, 84.2% to 91.3%), respectively. The comparison of individual events between patients with and without DM in the matched population is summarized in Table 3. Patients with DM had a significantly higher risk of cardiovascular death, in addition to all-cause mortality and cardiovascular events, than patients without DM; the hazard ratio of DM for cardiovascular death was 2.61 (95% CI, 1.16 to 5.87; *P* = 0.021). DM had no significant association with any other component of cardiovascular events (all *P* > 0.05).

**Table 2:** Baseline characteristics in matched population

	Non-DM patients	DM patients	Standardized difference (%)
Age (years)	75 [74 to 75]	75 [74 to 76]	2.7
Male sex	84.5% [81.8% to 87.1%]	84.2% [79.4% to 88.9%]	0.8
Smoking			
Never	22.3% [19.2% to 25.4%]	22.3% [16.8% to 27.7%]	0.4
Past	60.6% [57.0% to 64.2%]	59.6% [53.2% to 66.0%]	1.9
Current	17.1% [14.3% to 19.9%]	18.1% [13.1% to 23.1%]	2.6
Pack-years of smoking			
None	22.6% [19.4% to 25.7%]	22.5% [17.1% to 28.0%]	0.5
> 0 and ≤ 20 pack-years	11.6% [9.2% to 14.0%]	11.6% [7.0% to 16.3%]	2.4
> 20 and ≤ 40 pack-years	22.1% [18.9% to 25.4%]	22.5% [16.9% to 28.2%]	1.4
> 40 pack-years	43.7% [39.9% to 47.6%]	43.3% [36.7% to 49.9%]	1.7
BMI			
< 20 kg/m^2^	11.9% [9.5% to 14.3%]	11.8% [7.6% to 16.0%]	0.4
20 to 25 kg/m^2^	58.5% [54.9% to 62.2%]	57.9% [51.5% to 64.4%]	1.3
≥ 25 kg/m^2^	29.6% [26.2% to 32.9%]	30.3% [24.3% to 36.3%]	1.6
Hypertension	90.4% [88.2% to 92.5%]	91.0% [87.2% to 94.7%]	2.0
Dyslipidemia	85.6% [83.0% to 88.2%]	86.4% [82.0% to 90.9%]	2.4
Renal failure on dialysis	3.7% [2.3% to 5.1%]	4.5% [1.8% to 7.2%]	4.0
Chronic obstructive pulmonary disease	34.7% [31.2% to 38.2%]	33.9% [27.8% to 40.1%]	1.6
Malignant neoplasm			
None	73.7% [70.5% to 77.0%]	73.3% [67.5% to 79.1%]	1.0
Cured	15.2% [12.6% to 17.9%]	16.3% [11.5% to 21.1%]	2.9
In treatment	11.0% [8.7% to 13.4%]	10.4% [6.4% to 14.4%]	2.0
Family history of aortic aneurysm	3.7% [2.3% to 5.2%]	3.6% [1.0% to 6.3%]	2.2
Myocardial infarction	16.5% [13.8% to 19.3%]	17.6% [12.7% to 22.6%]	3.0
History of coronary revascularization	25.8% [22.5% to 29.0%]	26.7% [20.9% to 32.5%]	2.1
Cerebral hemorrhage	4.3% [2.8% to 5.8%]	4.1% [1.5% to 6.6%]	1.1
Cerebral infarction	13.9% [11.3% to 16.5%]	14.5% [9.9% to 19.1%]	1.8
LEAD	11.3% [8.7% to 13.9%]	13.5% [9.0% to 18.1%]	6.8
Statin use	56.6% [52.9% to 60.2%]	58.3% [51.9% to 64.8%]	3.5
Beta blocker use	39.9% [36.3% to 43.5%]	41.1% [34.7% to 47.5%]	2.4
Renin-angiotensin system inhibitor use	42.7% [39.0% to 46.4%]	41.7% [35.2% to 48.1%]	2.1
Antiplatelet use	48.5% [44.8% to 52.2%]	48.8% [42.2% to 55.4%]	0.9
Anticoagulant use	18.2% [15.3% to 21.1%]	18.2% [13.2% to 23.3%]	0.7
Systolic blood pressure			
< 120 mmHg	27.6% [24.3% to 30.9%]	27.2% [21.4% to 33.1%]	0.8
120 to 140 mmHg	48.0% [44.3% to 51.7%]	48.2% [41.7% to 54.8%]	0.6
140 to 160 mmHg	18.8% [15.9% to 21.7%]	18.2% [13.1% to 23.2%]	1.5
≥ 160 mmHg	5.6% [3.9% to 7.3%]	6.3% [3.2% to 9.5%]	3.2
Diastolic blood pressure			
< 80 mmHg	63.4% [59.8% to 67.0%]	63.3% [57.0% to 69.6%]	0.5
80 to 90 mmHg	22.6% [19.5% to 25.7%]	21.8% [16.4% to 27.2%]	2.1
90 to 100 mmHg	10.9% [8.6% to 13.2%]	11.8% [7.6% to 16.0%]	3.0
≥ 100 mmHg	3.1% [1.8% to 4.4%]	3.2% [0.9% to 5.5%]	0.5
Non-HDL cholesterol			
< 100 mg/dl (< 2.59 mmol/l)	24.8% [21.3% to 28.3%]	25.8% [19.9% to 31.7%]	2.7
100 to 130 mg/dl (2.59 to 3.36 mmol/l)	31.9% [28.2% to 35.7%]	31.5% [24.9% to 38.1%]	2.6
130 to 170 mg/dl (3.36 to 4.40 mmol/l)	31.1% [27.3% to 34.9%]	31.5% [24.9% to 38.1%]	2.8
≥ 170 mg/dl (≥ 4.40 mmol/l)	12.2% [9.6% to 14.8%]	11.1% [6.9% to 15.4%]	3.3
LDL cholesterol			
< 70 mg/dl (< 1.81 mmol/l)	15.3% [12.5% to 18.0%]	14.9% [9.9% to 19.9%]	2.4
70 to 100 mg/dl (1.81 to 2.59 mmol/l)	35.7% [32.1% to 39.3%]	37.3% [30.7% to 43.9%]	3.7
100 to 140 mg/dl (2.59 to 3.62 mmol/l)	36.6% [33.0% to 40.3%]	36.7% [30.4% to 43.0%]	0.7
≥ 140 mg/dl (≥ 3.62 mmol/l)	12.4% [9.9% to 14.9%]	11.1% [6.9% to 15.4%]	3.9
HDL cholesterol			
< 40 mg/dl (< 1.03 mmol/l)	28.6% [25.1% to 32.1%]	28.6% [22.3% to 34.9%]	2.1
40 to 50 mg/dl (1.03 to 1.29 mmol/l)	33.7% [29.8% to 37.5%]	34.8% [28.3% to 41.2%]	2.7
50 to 60 mg/dl (1.29 to 1.55 mmol/l)	18.6% [15.1% to 22.0%]	18.1% [12.8% to 23.5%]	2.1
≥ 60 mg/dl (≥ 1.55 mmol/l)	19.2% [16.0% to 22.3%]	18.5% [13.2% to 23.8%]	2.3
Triglycerides			
< 100 mg/dl (< 1.13 mmol/l)	31.3% [27.7% to 34.8%]	31.6% [25.4% to 37.8%]	1.3
100 to 150 mg/dl (1.13 to 1.69 mmol/l)	31.5% [28.0% to 35.0%]	32.1% [25.8% to 38.3%]	1.7
150 to 200 mg/dl (1.69 to 2.26 mmol/l)	21.6% [18.4% to 24.7%]	20.7% [15.2% to 26.1%]	2.6
≥ 200 mg/dl (≥ 2.26 mmol/l)	15.7% [12.9% to 18.4%]	15.7% [10.9% to 20.4%]	0.8
Estimated glomerular filtration rate			
< 15 ml/min/1.73 m^2^	5.0% [3.3% to 6.6%]	5.4% [2.5% to 8.4%]	2.1
15 to 30 ml/min/1.73 m^2^	6.9% [5.0% to 8.8%]	6.8% [3.5% to 10.1%]	0.4
30 to 60 ml/min/1.73 m^2^	49.4% [45.7% to 53.1%]	49.8% [43.3% to 56.3%]	0.7
≥ 60 ml/min/1.73 m^2^	38.7% [35.1% to 42.4%]	38.0% [31.7% to 44.3%]	1.5
LVEF			
< 50%	9.2% [7.0% to 11.4%]	9.3% [5.4% to 13.1%]	1.1
50% to 60%	17.8% [14.9% to 20.8%]	18.8% [13.6% to 24.0%]	2.5
60% to 70%	57.5% [53.5% to 61.4%]	57.0% [50.4% to 63.6%]	1.3
≥ 70%	15.5% [12.6% to 18.4%]	14.9% [10.1% to 19.8%]	2.2

Data in non-DM and DM patients are estimated means or proportions [95% confidence intervals] obtained from the multiple imputation

*BMI* Body mass index, *HDL* High-density lipoprotein, *LDL* Low-density lipoprotein, *LEAD* Lower extremity artery disease, *LVEF* Left ventricular ejection fraction

**Table 3:** Comparison of event incidence between DM and non-DM in matched population

	Incidence rate in non-DM patients (per 100 person-years)	Incidence rate in DM patients (per 100 person-years)	Hazard ratio [95% confidence interval] of DM versus non-DM (*P* value)
All-cause mortality	5.90	11.55	2.28 [1.37 to 3.81] (*P* = 0.002)
Cardiovascular event	12.43	19.85	1.67 [1.13 to 2.46] (*P* = 0.010)
Cardiovascular death	2.12	5.17	2.61 [1.16 to 5.87] (*P* = 0.021)
Aortic event	6.31	8.78	1.25 [0.73 to 2.16] (*P* = 0.41)
Aortic rupture	0.49	0.91	1.88 [0.34 to 10.4] (*P* = 0.47)
Endoleak	1.92	2.86	2.29 [0.91 to 5.74] (*P* = 0.077)
Graft occlusion	0.00	0.00	-
Graft infection	0.94	0.61	0.55 [0.11 to 2.85] (*P* = 0.48)
Graft migration	0.49	0.30	0.52 [0.05 to 5.25] (*P* = 0.58)
Sac enlargement	1.79	3.13	1.03 [0.40 to 2.69] (*P* = 0.94)
Reintervention	3.20	4.87	1.18 [0.54 to 2.56] (*P* = 0.68)
Cerebral event	3.15	4.72	1.41 [0.68 to 2.91] (*P* = 0.36)
Cerebral hemorrhage	0.41	1.83	2.35 [0.59 to 9.45] (*P* = 0.23)
Cerebral infarction	2.76	2.81	1.15 [0.48 to 2.74] (*P* = 0.75)
Cardiac event	2.38	3.73	1.80 [0.72 to 4.47] (*P* = 0.21)
Myocardial infarction	0.66	0.91	2.28 [0.43 to 12.1] (*P* = 0.33)
Hospitalization for heart failure	1.70	2.80	1.63 [0.55 to 4.82] (*P* = 0.38)
Lower extremity event	0.44	0.91	2.91 [0.44 to 19.1] (*P* = 0.27)
Limb-threatening ischemia	0.42	0.91	4.37 [0.44 to 43.1] (*P* = 0.21)
Major amputation	0.02	0.00	-

Hyphens indicate that Cox regression models were not converged and hazard ratios were not calculated

In patients with DM, plasma glucose levels were significantly associated with cardiovascular events (*P* = 0.032); the hazard ratio was 1.08 (95% CI, 1.01 to 1.16) per 1-mmol/L increase. On the other hand, plasma glucose levels were not significantly associated with all-cause mortality (*P* = 0.36). Neither were HbA1c levels significantly associated with cardiovascular events or all-cause mortality (*P* = 0.46 and 0.59) (Additional file 1: Tables S3). Increased BMI was inversely associated with the risk of all-cause mortality and cardiovascular event; the hazard ratio of BMI ≥ 25 kg/m^2^ versus < 20 kg/m^2^ was 0.40 (95% CI, 0.18 to 0.87; *P* = 0.021) and 0.49 (95% CI, 0.26 to 0.91; *P* = 0.024), respectively. The association did not differ between non-DM patients and DM patients (P for interaction = 0.82 and 0.29, respectively) (Additional file 1: Tables S4).

## Discussion

The current study compared the baseline characteristics and prognoses of patients with and without DM undergoing endovascular AA repair. Patients with DM had higher pack-years of smoking, higher BMI, lower HDL cholesterol levels, higher triglyceride levels, and lower LVEF than those without DM, whereas the non-HDL and LDL cholesterol levels and BP did not differ between the groups. Patients with DM also had a high prevalence of coronary artery disease and LEAD. After endovascular repair, DM patients had a higher incidence of all-cause mortality and cardiovascular events. Their poorer prognoses were further confirmed when the baseline characteristics were matched. To the best of our knowledge, this is the first study to compare smoking amount, BP control, and lipid profiles between DM and non-DM patients with AA, and to compare prognoses after repair with adjustment for these detailed clinical profiles.

Peripheral artery disease encompasses a range of noncoronary arterial syndromes that are caused by the altered structure and function of arteries [24]. Though AA is included, it is distinct from many other entities of peripheral artery disease because AA is a localized dilation of an arterial segment, while others are often occlusive. Epidemiological studies have shown that most risk factors for atherosclerotic occlusive diseases also increase the risk of incident AA, but one marked exception is DM [25]. Patients with DM are less likely to develop AA than those without DM. Why DM protects against AA remains to be fully elucidated. One suggested explanation is that DM promotes extracellular matrix formation and reduces its degradation [26]. Such changes in matrix remodeling may protect DM patients from the risk of developing AA. It was also reported that DM patients had greater intima-media thickness in the aorta, mitigating aortic wall stress, compared with non-DM patients [27]. Nonetheless, there are some DM patients in clinical practice who develop AA, despite the potent protection by DM.

A simple speculation concerning AA development in DM patients is that DM patients who develop AA might be more heavily exposed to major risk factors for AA, such as smoking. However, previous studies often failed to show a clear difference in smoking status between DM and non-DM patients with AA [13–15], which is consistent with the current findings. In contrast, we confirmed that the amount of smoking, measured by pack-years, had a significant intergroup difference, with DM patients having higher pack-years of smoking. Smoking, well recognized as a leading cause of AA, increases the risk of AA development in a dose-dependent manner [6]. Therefore, increased exposure may negate the protective effects of DM.

On the other hand, LDL or non-HDL cholesterol levels and BP, which are factors associated with incident AA [6–9], were not significantly different between patients with and without DM. One possible explanation is that these profiles were measured only during AA repair, and the profiles might have been previously uncontrolled. More frequent statin treatment, a more common history of coronary artery disease and LEAD, and lower LVEF indicating impaired cardiac function, might be suggestive of past exposure to uncontrolled LDL or non-HDL cholesterol levels and BP in DM patients.

Our study also revealed that DM patients had higher BMI, lower HDL cholesterol, and higher triglyceride levels. The impact of BMI, HDL cholesterol levels, and triglyceride levels on the risk of AA is controversial [6, 8, 28–31]. The higher levels in DM patients might simply reflect the fact that DM, obesity, reduced HDL cholesterol levels, and hypertriglyceridemia, being components of metabolic syndrome, are likely accumulated [32], rather than suggesting that obesity, reduced HDL cholesterol levels, and hypertriglyceridemia per se counteract the protective effects of DM against AA development.

Thus, in a population undergoing endovascular AA repair, patients with DM had more cardiovascular risk factors than those without DM. These cardiovascular risk factors are generally associated with poor prognoses in many other populations [24, 33–35]. Therefore, our crude prognostic comparisons, which demonstrated poorer prognosis of DM patients with AA might be unsurprising. However, interestingly, DM patients still had a higher incidence rate of mortality and cardiovascular events after matching for baseline characteristics including these risk factors. DM per se would be a risk factor for poor prognosis after AA repair. It would be of clinical importance to identify a subgroup at high risk of poor prognosis in patients with DM. 

The present study showed a significant association between plasma glucose levels and cardiovascular events in patients with DM, potentially suggesting that DM patients with poor glycemic control would have a higher risk of incident cardiovascular events. However, HbA1c levels were not significantly associated with cardiovascular events. Neither were plasma glucose or HbA1c levels significantly associated with all-cause mortality. The non-significance in these associations might come from insufficient statistical power (the present study observed 40 mortalities and 59 cardiovascular events in 226 DM patients); their associations would be statistically inconclusive. Another explanation for the finding that glycemia but not HbA1c was associated with cardiovascular events might be that HbA1c levels are interfered with by kinetics of hemoglobin, which might mask a potential association of poor glycemic control with poor prognoses. Several studies reported the association of glycemic control and duration of DM with prognoses after aortic aneurysm repair [18, 19, 36]. Hjellestad and colleagues reported that HbA1c was an independent predictor for all-cause mortality in 66 DM patients with abdominal aortic aneurysms admitted to the vascular surgery unit for elective surgery [36]. On the other hand, Taimour and colleagues reported that in 748 DM patients undergoing elective endovascular aortic aneurysm repair, HbA1c levels and duration of DM were not significantly associated with all-cause mortality, cardiovascular mortality, acute myocardial infarction, or stroke [18]. They also reported in another study that in 363 type 2 DM patients undergoing acute aortic aneurysm repair, HbA1c levels and duration of DM were not significantly associated with all-cause mortality, cardiovascular mortality, major adverse cardiovascular events, or acute myocardial infarction, but that a higher HbA1c level was narrowly associated with incident stroke [19]. Future larger studies will be needed to validate the association of these parameters with prognoses.

The current registry did not collect data on other DM-related factors including type of DM, years of diagnosis, antidiabetic treatment, insulin resistance, and beta-cell dysfunction, which would potentially explain an increased risk of poor prognoses in DM patients. The contribution of those DM-related factors remained unknown. We supplementarily analyzed the prognostic impact of BMI, generally referred to as a surrogate marker of insulin resistance (but not a direct measurement). Consequently, its impact in DM patients did not different from that in matched non-DM patients. Given that BMI was balanced in the matched population, BMI was unlikely to explain the excess risk of poor prognoses in DM patients. BMI was rather inversely associated with the risk of all-cause mortality and cardiovascular event, suggesting the obesity paradox [37–39].

There are several limitations in the current study. First, as aforementioned, important information related to DM, including type of DM, duration of DM (or year of diagnosis), insulin use, other antidiabetic medications, insulin resistance, and beta-cell dysfunction, was not collected in the EOLIA registry. Data on insulin levels were also unavailable, and indices of insulin resistance and beta-cell dysfunction were unable to be calculated. The involvement of those DM-related factors in the association between DM and a poor prognosis remained unrevealed. Second, this study only enrolled Japanese patients who underwent endovascular AA repair. It remains to be determined whether similar findings were observed in other populations, such as other ethnic groups and those undergoing open surgical repair. Third, data were not available on clinical profiles and medication use before AA development and after AA repair. These unmeasured variables might be different between DM and non-DM patients, and might interfere with the prognostic differences between the two populations. Fourth, the follow-up period was limited. Future studies investigating longer-term prognosis are needed.

## Conclusions

Among patients undergoing endovascular repair of AA, DM patients had higher pack-years of smoking, higher BMI, lower HDL cholesterol levels, higher triglyceride levels, and lower LVEF than non-DM patients. Meanwhile, the LDL or non-HDL cholesterol levels and BP did not differ between the groups. Patients with DM also had a higher prevalence of coronary artery disease and LEAD. After AA repair, patients with DM had a higher risk of all-cause mortality and cardiovascular events. A poorer prognosis was confirmed even after matching for baseline characteristics.

## Supplementary Information

**Additional file 1.**

## Data Availability

The datasets generated and/or analyzed during the current study are not publicly available due to ethical reasons but are available from the corresponding author upon reasonable request.

## Abbreviations

AA: Aortic aneurysm
BMI: Body mass index
BP: Blood pressure
CI: Confidence interval
DM: Diabetes mellitus
EOLIA: Efficacy and safety of endovascular repair for abdominal and thoracic aortic aneurysms
HDL: High-density lipoprotein
LDL: Low-density lipoprotein
LEAD: Lower extremity artery disease
LVEF: Left ventricular ejection fraction
SD: Standard deviation
