# Supplementary material for: Clinical features and prognosis of patients with and without diabetes mellitus undergoing endovascular aortic aneurysm repair
Source: BMC Endocr Disord. 2022 Apr 7;22:92. doi: 10.1186/s12902-022-01008-4 (PMC8988424; doi:10.1186/s12902-022-01008-4)
Supplement: Supplementary file 1 — Additional file 1. [file 12902_2022_1008_MOESM1_ESM.docx]

**Additional file 1: Table S1. Ambulatory status, aortic lesion characteristics, and procedures in the overall population**

|  | Overall population | Missing data | Non-DM patients  (n = 703) | DM patients  (n = 226) | P value |
| --- | --- | --- | --- | --- | --- |
| Ambulatory status |  |  |  |  | 0.66 |
| Ambulatory | 848 (91.3%) |  | 90.6% [88.5% to 92.8%] | 93.4% [90.1% to 96.6%] |  |
| In wheelchair | 54 (5.8%) |  | 6.8% [5.0% to 8.7%] | 2.7% [0.6% to 4.8%] |  |
| Bed-ridden | 27 (2.9%) |  | 2.6% [1.4% to 3.7%] | 4.0% [1.4% to 6.5%] |  |
| Thoracic aortic aneurysm | 321 (34.6%) |  | 33.3% [29.8% to 36.8%] | 38.5% [32.2% to 44.8%] | 0.18 |
| Abdominal aortic aneurysm | 616 (66.3%) |  | 67.4% [64.0% to 70.9%] | 62.8% [56.5% to 69.1%] | 0.23 |
| Complication with iliac aneurysm | 112 (12.1%) |  | 12.4% [9.9% to 14.8%] | 11.1% [7.0% to 15.2%] | 0.68 |
| Aneurysm diameter (mm) | 52 ± 11 | 1 (0.1%) | 52 [51 to 53] | 53 [52 to 55] | 0.31 |
| General anesthesia | 774 (83.3%) |  | 83.1% [80.3% to 85.8%] | 84.1% [79.3% to 88.8%] | 0.80 |
| Femoral approach | 893 (96.1%) |  | 96.9% [95.6% to 98.2%] | 94.2% [91.2% to 97.3%] | 0.11 |
| Iliac artery as distal landing zone | 589 (64.4%) | 14 (1.5%) | 65.9% [62.4% to 69.4%] | 61.5% [55.2% to 67.8%] | 0.26 |
| Side branch intervention | 441 (47.7%) | 5 (0.5%) | 47.5% [43.8% to 51.2%] | 48.5% [42.0% to 55.0%] | 0.86 |
| Debranching | 87 (9.4%) |  | 8.7% [6.6% to 10.8%] | 11.5% [7.3% to 15.7%] | 0.26 |
| Spinal drainage | 37 (4.0%) |  | 4.3% [2.8% to 5.8%] | 3.1% [0.8% to 5.4%] | 0.56 |
| Procedure time (min) | 142 ± 73 | 3 (0.3%) | 142 [137 to 147] | 142 [131 to 153] | 0.99 |
| Contrast volume (ml) | 96 ± 48 | 13 (1.4%) | 97 [93 to 100] | 94 [88 to 100] | 0.52 |

Data in non-DM and DM patients are estimated means or proportions [95% confidence intervals] obtained from the multiple imputation.

**Additional file 1: Table S2. Ambulatory status, aortic lesion characteristics, and procedures in the matched population**

|  | Non-DM patients | DM patients | Standardized difference (%) |
| --- | --- | --- | --- |
| Ambulatory status |  |  |  |
| Ambulatory | 93.3% [91.5% to 95.2%] | 93.2% [89.9% to 96.5%] | 0.5 |
| In wheelchair | 2.7% [1.5% to 3.8%] | 2.7% [0.6% to 4.8%] | 0.4 |
| Bed-ridden | 4.0% [2.6% to 5.5%] | 4.1% [1.5% to 6.6%] | 0.3 |
| Thoracic aortic aneurysm | 36.4% [32.8% to 39.9%] | 37.6% [31.2% to 43.9%] | 2.4 |
| Abdominal aortic aneurysm | 64.4% [60.8% to 67.9%] | 63.3% [57.1% to 69.6%] | 2.1 |
| Complication with iliac aneurysm | 11.7% [9.4% to 14.1%] | 11.3% [7.2% to 15.4%] | 1.3 |
| Aneurysm diameter (mm) | 53 [52 to 54] | 53 [51 to 55] | 0.3 |
| General anesthesia | 84.2% [81.5% to 86.9%] | 84.6% [79.9% to 89.3%] | 1.1 |
| Femoral approach | 94.8% [93.1% to 96.4%] | 95.0% [92.2% to 97.9%] | 1.2 |
| Iliac artery as distal landing zone | 62.9% [59.3% to 66.5%] | 62.0% [55.7% to 68.3%] | 1.9 |
| Side branch intervention | 48.2% [44.5% to 51.9%] | 48.2% [41.7% to 54.8%] | 0.4 |
| Debranching | 11.3% [9.0% to 13.6%] | 10.9% [6.8% to 14.9%] | 1.4 |
| Spinal drainage | 2.9% [1.6% to 4.1%] | 3.2% [0.9% to 5.5%] | 1.7 |
| Procedure time (min) | 142 [137 to 147] | 142 [131 to 153] | 0.4 |
| Contrast volume (ml) | 96 [93 to 100] | 94 [88 to 101] | 4.9 |

Data in non-DM and DM patients are estimated means or proportions [95% confidence intervals] obtained from the multiple imputation.

**Additional file 1: Table S3. Association of glycemic control with the risk of all-cause mortality and cardiovascular event in DM patients**

|  | All-cause mortality | Cardiovascular event |
| --- | --- | --- |
| Plasma glucose (per 1-mmol/L increase) | 1.04 [0.95 to 1.14] (P=0.36) | 1.08 [1.01 to 1.16] (P=0.032) |
| HbA1c (per 1% increase) | 0.85 [0.54 to 1.32] (P=0.46) | 0.90 [0.61 to 1.33] (P=0.59) |

Data are hazard ratios [95% confidence intervals] (P values).

**Additional file 1: Table S4. Association of BMI with the risk of all-cause mortality and cardiovascular event in matched non-DM and DM patients**

|  | Overall population | Non-DM patients | DM patients | P for interaction |
| --- | --- | --- | --- | --- |
| All-cause mortality |  |  |  |  |
| BMI < 20 kg/m^2^ | 1.00 (Reference) | 1.00 (Reference) | 1.00 (Reference) | P = 0.82 |
| BMI ≥ 20 and < 25 kg/m^2^ | 0.51 [0.27 to 0.96] (P=0.037) | 0.50 [0.24 to 1.05] (P=0.069) | 0.44 [0.11 to 1.73] (P=0.24) |  |
| BMI ≥ 25 kg/m^2^ | 0.40 [0.18 to 0.87] (P=0.021) | 0.41 [0.16 to 1.06] (P=0.065) | 0.24 [0.05 to 1.14] (P=0.073) |  |
| Cardiovascular event |  |  |  |  |
| BMI < 20 kg/m^2^ | 1.00 (Reference) | 1.00 (Reference) | 1.00 (Reference) | P = 0.29 |
| BMI ≥ 20 and < 25 kg/m^2^ | 0.74 [0.43 to 1.29] (P=0.29) | 0.59 [0.32 to 1.08] (P=0.085) | 1.53 [0.41 to 5.77] (P=0.53) |  |
| BMI ≥ 25 kg/m^2^ | 0.49 [0.26 to 0.91] (P=0.024) | 0.48 [0.24 to 0.95] (P=0.035) | 0.69 [0.16 to 2.94] (P=0.61) |  |

Data are hazard ratios [95% confidence intervals] (P values).
